# Assessment of a variety of dispersion-corrected density functional theory calculations used in molecular crystal structure prediction

**DOI:** 10.1186/1758-2946-4-S1-O15

**Published:** 2012-05-01

**Authors:** Bernd Doser, Marcus A Neumann

**Affiliations:** 1 Avant-garde Materials Simulation, Merzhauser Straße 177, 79100 Freiburg im Breisgau, Germany

## 

For a long time, the prediction of molecular crystal structures from first principles has been one of the most challenging computational problems. Because of the large number of degrees of freedom, methods with low computational effort have to be used. The price for this efficiency is, however, a loss of accuracy. In recent years, important progress has been made in the development of dispersion corrections [1-3], which raise the accuracy of density functional theory (DFT) calculations to a much higher level.

Based on these developments, the software package GRACE [4] was the first program ever to correctly predict all four crystal structures at the 4th crystal structure prediction blind test, organized by G. M. Day and the Cambridge Crystallographic Data Centre in 2007 [5]. GRACE uses dispersion-corrected density functional theory calculations (DFT-D) to generate reference data to which a tailor-made force field (TMFF) is fitted for each respective molecule [6]. Crystal structures are generated with a Monte-Carlo parallel-tempering algorithm that uses the TMFF for the evaluation of lattice energies. Only the most stable crystal structures according to the TMFF are then re-optimized and re-ranked using DFT-D. The DFT calculations are performed using an interface to the two common ab-inito programs VASP [7] and QuantumESPRESSO [8].

In the most recent, 5th blind test, organized in 2010, the excellent agreement between experimental and predicted structures for small, neutral molecules was confirmed. However, it turned out that the crystal structure prediction of molecular salts and hydrates is still a challenging task. The inability of DFT without exact exchange to describe anions properly, as recently published by Jensen [9], suggests that the inclusion of the Hartree-Fock exchange should improve the accuracy for molecular salts and hydrates. Therefore, we will present an assessment of a variety of current density functionals, in which the dispersion-correction parameters were fitted with respect to a minimization of the deviation between experimental and computed crystal structures.
